# Embryogenesis of the First Circulating Endothelial Cells

**DOI:** 10.1371/journal.pone.0060841

**Published:** 2013-05-30

**Authors:** Cheng Cui, Michael B. Filla, Elizabeth A. V. Jones, Rusty Lansford, Tracey Cheuvront, Sarah Al-Roubaie, Brenda J. Rongish, Charles D. Little

**Affiliations:** 1 Department of Anatomy and Cell Biology, University of Kansas Medical Center, Kansas City, Kansas, United States of America; 2 Department of Chemical Engineering, McGill University, Montreal, Canada; 3 Beckman Institute, California Institute of Technology, Pasadena, California, United States of America; Peking University, China

## Abstract

Prior to this study, the earliest appearance of circulating endothelial cells in warm-blooded animals was unknown. Time-lapse imaging of germ-line transformed Tie1-YFP reporter quail embryos combined with the endothelial marker antibody QH1 provides definitive evidence for the existence of circulating endothelial cells – from the very beginning of blood flow. Blood-smear counts of circulating cells from Tie1-YFP embryos showed that up to 30% of blood-borne cells are Tie1 positive; though cells expressing low levels of YFP were also positive for benzidine, a hemoglobin stain, suggesting that these cells were differentiating into erythroblasts. Electroporation-based time-lapse experiments, exclusively targeting the intra-embryonic mesoderm were combined with QH1 immunostaining. The latter antibody marks quail endothelial cells. Together the optical data provide conclusive evidence that endothelial cells can enter blood flow from vessels of the embryo proper, as well as from extra-embryonic areas. When Tie1-YFP positive cells and tissues are transplanted to wild type host embryos, fluorescent cells emigrate from such transplants and join host vessels; subsequently a few YFP cells are shed into circulation. These data establish that entering circulation is a commonplace activity of embryonic vascular endothelial cells. We conclude that in the class of vertebrates most closely related to mammals a normal component of primary vasculogenesis is production of endothelial cells that enter circulation from all vessels, both intra- and extra-embryonic.

## Introduction

Endothelial cells that circulate in the peripheral blood are a heterogeneous population, consisting of both mature endothelial cells, which are believed to have sloughed off the vessel wall, and of bone marrow derived progenitors, which play a role in vascularization [1]. Ashara and colleagues (1997) labeled circulating endothelial progenitors that incorporated into the vessel wall of new capillaries in a hindlimb ischemia model [2]. Similar results have been found in many other models including retinal vascularization [3], tumor angiogenesis [4], and wound healing [4]. Clinical trials are underway using circulating endothelial cells derived from bone marrow or from peripheral blood, to treat acute myocardial ischemia [5], [6], chronic coronary total occlusion [7] and non-ischemic cardiomyopathy [8].

The origin of circulating endothelial cells during vascular development is unresolved, in amniotes. In the adult, circulating endothelial cells capable of participating in vascularization originate from the bone marrow [9]. The first site(s) of hematopoiesis during embryogenesis occur at structures referred to as blood islands. Indeed, just as with the adult hematopoietic organ, embryonic blood islands produce endothelial cells that enter circulation. Labeling of the blood islands with a virus encoding a fluorescent protein, expressed before the onset of circulation, results in the presence of fluorescent endothelial cells throughout the embryonic vascular plexus [10]. However, there are no direct imaging studies confirming the existence of circulating endothelial cells in peripheral blood during early bird or mammalian embryogenesis. There is ample evidence that extra-embryonic blood islands can produce circulating endothelial cells; however, the potential of intra-embryonic endothelial tubes/clusters to produce circulating endothelial cells remains contentious [10], [11], [12]. Caprioli *et al*. [11] demonstrated that the allantois, an extra-embryonic tissue, was a source both of hematopoietic and endothelial cells and that these cells were able to colonize the bone marrow later in development. Pardanaud and Eichmann used chimeras of quail and chicken embryos to show that circulating quail endothelial cells can populate chicken blood vessels [13]. However, when they repeated these experiments using quail embryos devoid of extra-embryonic membranes, they did not observe quail endothelial cells integrating into chicken blood vessels.

The integration of circulating endothelial cells into blood vessel walls appears to be a very rare event both in adults and during vascular development. In some models of adult angiogenesis, induced by vascular endothelial growth factor (VEGF), bone marrow-derived smooth muscle, pericytes and leukocytes are present in the nascent vasculature area but never bone marrow-derived endothelial cells [14], [15]. Using the embryonic model of chick-quail parabiosis – an experimental system whereby a chicken and a quail embryo share a single chorioallantoic membrane – Pardanaud and Eichmann found that one day after circulation between the two systems was established, few quail endothelial cells were present (3 to 6 endothelial cells per mm^3^) [13]. Similar to the adult, VEGF-induced vascularization in the embryonic chorioallantoic membrane did not increase the integration of circulating cells into the newly formed blood vessels. The authors suggested that circulating endothelial cells are important during vasculogenesis but not when vessels form via sprouting [13]. Prior to this study, the abundance of endothelial cells circulating in the blood of a vasculogenic-stage amniote embryo was unknown – despite a host of studies that focused on the question of how endothelial cells integrate into blood vessels during development.

To address directly the spatial and temporal origin of circulating endothelial cells, and their subsequent behavior, we employed three strategies: 1) We recorded live transgenic quail embryos, which express yellow fluorescent protein in endothelial (and endocardial) cells under the control of the marker gene *tie1*. Tie1 is also expressed in early hematopoietic stem cells [16]. Observing *tie1* embryos we discovered abundant circulating fluorescent endothelial cells. In fact up to 30% of blood-borne cells during the early phases of fluid flow expressed Tie1-YFP. Circulating Tie-1 positive cells expressing low levels of YFP were benzidine positive, indicating the presence of hemoglobin. Surprisingly a number of tagged cells appeared to arise directly from the intra-embryonic lateral mesoderm; although the majority of the circulating YFP-positive cells arose from extra-embryonic tissue. 2) To confirm the possibility that circulating endothelial cells could arise within the embryo proper, we performed targeted electroporation of specimens at Hamburger and Hamilton stage 4-minus (HH4-, [17]) with DNA plasmids encoding a fluorescent protein – thereby labeling intra-embryonic mesoderm, and only intra-embryonic mesoderm. Electroporated embryos were then co-labeled with endothelial specific marker QH1. The electroporation experiments yielded the first data demonstrating directly that primordial vessels within the soma of an embryonic amniote shed endothelial cells into circulation. 3) We tested whether transplanted Tie1-YFP endothelial cells would shed into circulation when grafted into a wild type embryo. Tie1-YFP cells were observed in circulation after transplantation of tail bud tissue, dispersed cells or blood-borne cells, into wild type embryos. The transplantation data confirm that shedding into circulation is a general property of embryonic endothelial cells.

The ability to record the behavior of circulating Tie-1 positive cells, *in situ,* using the transgenic quail embryos also allowed us to observe the various journeys of such cells during early vascular development throughout an entire specimen (n = 105). Three general behaviors were observed: 1) some fluorescent circulating Tie-1 positive cells move freely with blood flow, 2) a fraction of the cells roll along the luminal surface of the endothelium at very low speeds and 3) some tagged cells move in a jerky start-and-stop, or saltatory, manner. Blood vessels of virtually all sizes and from all anatomical regions shed Tie-1 positive cells into circulation.

### Operational Definitions

*Rapid displacement behavior* – The case where a fluorescent cell present in one time-lapse frame ‘disappears’ from view or shifts to a distinctly new XY position within one time-lapse interval.

*Saltatory Behavior* – The case of cells or cellular clusters being moved, by fluid flow, in an abrupt start-and-stop fashion, rather than by smooth gradual displacements.

## Results

### Circulating Tie-1 Positive Cells Appear as soon as Fluid Flow Is Initiated

**Figure 1:**
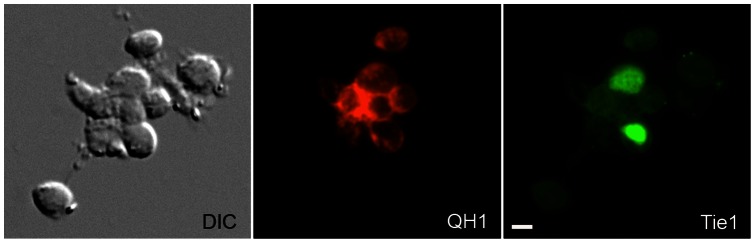
Double fluorescence imaging of adherent cells isolated from embryonic blood. Cells attached to a culture surface after being harvested from the circulating blood of a HH15 transgenic quail embryo. The green nuclei denote *tie1-H2B-YFP* expression. The red signal denotes cells reactive with the QH1 antibody. Under the conditions employed, quail erythrocytes (red blood cells) adhered poorly to the culture surface. Differential interference contrast optics (DIC) showed that there are adherent cells present that do not exhibit fluorescence above background levels. Scale bar  = 10 µm.

**Figure 2:**
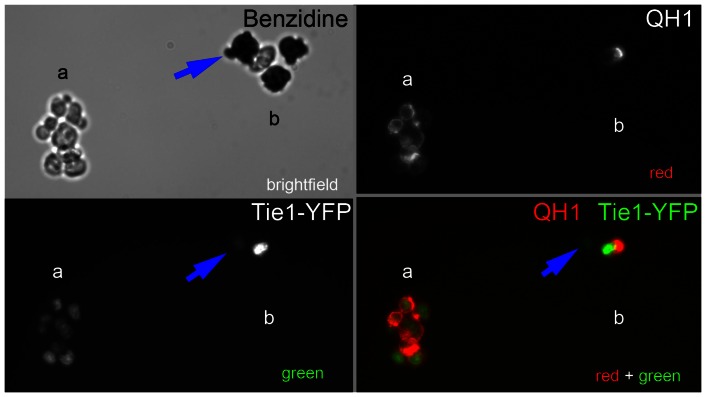
Benzidine staining of circulating whole blood. Fertilized quail eggs were incubated approximately 50 hours until they had reached between HH15 and HH17. Circulating cells were isolated and stained for QH1 that labels endothelial cells and benzidine that indicates the presence of hemoglobin. Cells expressing low levels of Tie1-YFP (blue arrow) were found to be QH1 negative and benzidine positive, suggesting that the cells were differentiating into erythroblasts. Scale bars  = 30 µm.

Transgenic quails that bear a nuclear-localized fluorescent marker driven by the *Tie1* promoter (Fig. 1 and 2), *Tg(tie1:H2B::eYFP)*, were used to examine endothelial cell motion during early embryogenesis [18]. Surprisingly large numbers of YFP-labeled cells were present in circulation after the onset of blood flow. We recorded a typical *Tie1-YFP* embryo at one frame per second (fps; Movie S1). At this frame rate, most, but not all, circulating Tie1-YFP cells moved at the speed of blood flow, with the fastest fluorescent circulating cells appearing as bright objects ‘streaking’ through vascular lumens. Freezing the motion of circulating RBCs would require frame rates approaching 100 fps. Movie S1 shows evidence for thousands of circulating Tie1-YFP cells flowing through the embryonic vasculature, at stages HH12-18. To visualize and appreciate the motion of individual fluorescent cells, it is necessary for readers to use software that supports frame-by-frame progression through the time-lapse sequence, such as QuickTime Player^TM^. Frame-by-frame analysis allows visualization of both the ‘streaks’ and perception of a sub-set of Tie1-YFP cells that are moving slower than blood flow (e.g., circled object Movie S1 at 10.8–10.9 min).

To determine where and when Tie1-YFP cells enter circulation we examined blood flow in an entire embryo. This was accomplished using wide-field acquisiton that permitted simultaneous recording of both differential interference contrast (DIC) and fluorescence images via time-lapse microscopy (n = 105). In all specimens a sub-population of Tie1-YFP cells was observed to exit a visual region of interest (ROI) during the interval between two consecutive frames. Numerous examples are apparent within the ROI denoted by the white box in Movie S2. Even more examples are visible in Movie S3 where a ROI is shown at three levels of magnification. A chevron at the 17.0 hr time point in Movie S3 indicates hundreds of Tie1-YFP cells that are flowing from caudal positions toward the inflow tract of the heart. At the relatively slow capture rates used, typically 6–12 minutes/frame, the rapid displacement behavior appears as a ‘flickering’ phenomenon. The onset of the rapid displacement behavior on the part of the Tie1-YFP cells always coincides with HH10, the stage at which heartbeat and fluid flow are initiated in quails (see the Introduction section for an operational definition of ‘rapid displacement behavior’). The exit of Tie1-YFP cells from a ROI cannot be accounted for by motion in the Z axis, because full-thickness Z planes were captured during each recording cycle (see Methods). The speed of normal endothelial cell motility is in the range of 0.01 µm/sec [19]. Given the size of a plausible ROI (XY = 1000 µm×1000 µm) an individual cell in the center of the ROI would have to move approximately 100 times faster than a typical cell (∼ = 0.7 µm/sec to ∼ = 1.0 µm/sec) in order to escape the ROI in one time-lapse interval (6-12 min) – a physical impossibility. A consequence of the prevailing biophysical conditions is that the fastest moving (flowing) Tie1-YFP cells are not easily perceived in Movies S2, and S3 (nor Movies S4, S6, S7, S9, S11). Overall, the wide-field imaging results indicate that Tie1-YFP cells enter circulation from locations throughout the entire embryonic and extra-embryonic vasculature concomitant with the onset of productive fluid flow (≈HH10); a process that accelerates markedly as fluid shear forces increase during stages HH11 to HH14, due to faster blood flow. See Movie Legends S2 and S3 for details of individual frames that demonstrate rapid displacement behavior.

**Figure 3:**
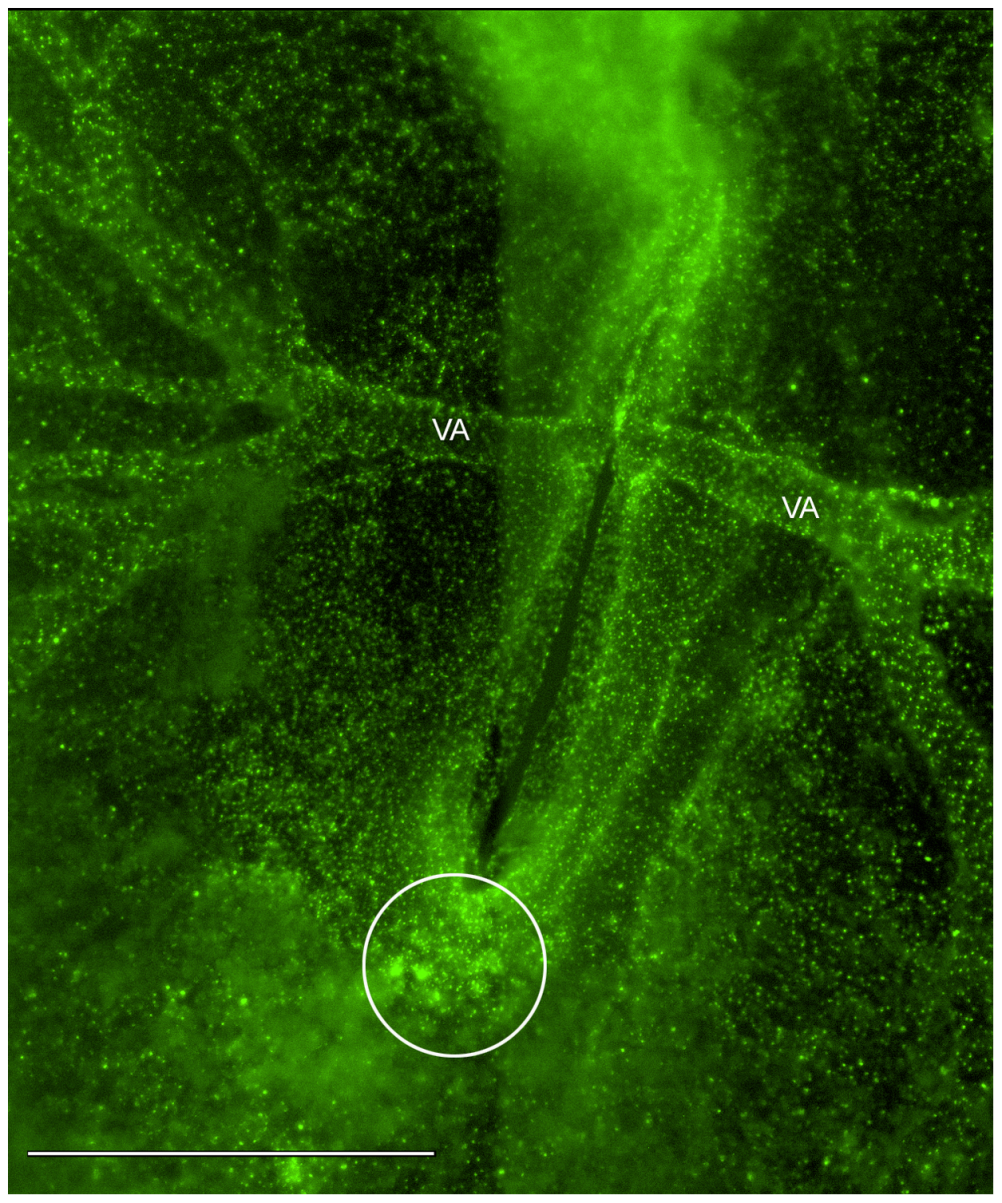
A montage of six image fields showing the vasculature of a Tie1-YFP embryo. This is a wide-field image frame extracted from a time-lapse recording of the Tie1-YFP transgenic quail embryo shown in Movie S3. Details regarding optics, instrumentation and signal processing are found in Methods and Materials. The fluorescent nuclear (H2B) staining denotes the position of all endothelial cells. The vitelline arteries (VA) are prominent blood vessels at HH15-16. The tail bud mesoderm (circle) shows numerous clusters of primordial endothelial cell nuclei that express the Tie1-YFP marker. Several movies in this report, and recordings not shown, demonstrate that ‘immobile’ clusters of primordial endothelial cells are gradually dispersed by the onset of fluid, flow and subsequently enter circulation. Thus, multi-cellular aggregates are a prominent source of circulating endothelial cells during primary vasculogenesis. The image shown is from Movie S3 at the 16.8 hr time point. Scale bar  = 1 mm or 1000 µm.

Simple inspection of wide-field recordings (n = 105) revealed that most circulating Tie1-positive cells originate from cellular aggregates/clusters (circle Fig. 3; also the large circle at 10 h in Movie S3). As productive heartbeat initiates fluid flow, nascent vascular channels gradually began conducting fluid in the spaces around the cellular clusters as shown in Movie S3 and in the ROI Movie S2. The forces of fluid flow appeared to shear fluorescent cells from the periphery of the Tie1-YFP clusters, particularly in the tail bud (circle, 10 h, Movie S3), after which the loosened cells and small clumps were swept away (chevrons, 17 h, Movie S3). This shedding process continued until a given cluster was eradicated. The erosion of Tie1-YFP cellular clusters from tail bud mesoderm continued throughout the circulation stages addressed in this study (HH10-18). Occasionally small clumps of Tie1-YFP cells moved as a unit in the bloodstream as shown when viewing Movie S1 frame-by-frame between 10.8 min to 10.9 min.

### Circulating Tie1-positive Cells are Abundant Early in Development

We next sought to quantify the proportion of Tie1-YFP cells in free circulation. At HH10-11 there is insufficient cardiac output to produce consistent physiological fluid flow. Productive circulation begins at HH11-13 as observed by stereomicroscope inspection. Accordingly, we used micro-techniques to harvest circulating blood beginning during the 2^nd^ day of incubation at HH14 until late in the 3^rd^ day of incubation at HH18. Depending on the stage, between 2–25 µl samples were removed from Tie1-YFP embryos, applied to a hemocytometer and photographed using fluorescence and DIC optics to determine the proportion of fluorescent cells (n = 8 embryos, 50 random hemocytometer fields). The percentage of Tie1-YFP cells varied considerably from a high of 30% at HH14 to 3.6% at HH18 (Table 1).

**Table 1:** Average proportion of Tie1 positive cells present in blood collected at various stages of development.

Embryo	HH Stage	Percent Tie1+ Cells in Blood (± Std. Error)
1	14	30.9±3.2%
2	15	24.5±6.9%
3	15	9.5±1.6%
4	15	12.6±2.4%
5	16	5.0±0.7%
6	18	4.0±0.8%
7	18	3.6±0.6%
8	18	8.5±1.6%
9	18	4.7±0.4%

Tie1 expression is widely acknowledged as an endothelial and endocardial lineage marker; early hematopoietic stem cells, however, are also known to express Tie1 [16]. In order to examine this possibility, we stained whole blood using benzidine, a stain that reacts with the pigment in hemoglobin (Fig. 2); it must be noted, however, that the benzidine reaction progressively quenches the Tie1-YFP fluorescence. Accordingly, blood samples from a clutch of embryos (HH15-17; n = 4) were divided equally. One aliquot showed that 15.5% of circulating cells were Tie-1 positive (±2%). The identical sister aliquot (n = 4) showed that most cells reacted positively with benzidine (>96%); thus, approximately 3% of cells failed to show a benzidine reaction (±0.8%).

Although detection of YFP fluorescence is compromised when using the benzidine reaction, we found that allowing Tie1-YFP cells to attach to a substrate permitted adequate detection of benzidine staining before substantial loss of the YFP signal (Fig. 2). Adherent cells, which expressed low levels of Tie1-YFP, were uniformly benzidine positive – the same cells, however, were negative for the quail marker antibody QH1. In contrast, strongly fluorescent Tie1-YFP cells were uniformly QH1 positive (Fig. 2).

To explore further the identity of the Tie1-YFP positive cells that failed to react with benzidine, we examined adherent blood-borne cells using various antibodies that recognize leukocyte and hematopoietic markers (HH12-20). Tie1-YFP cells were probed with an antibody for the leukocyte marker CD45. None of the Tie1-YFP-positive cells reacted with CD45, although a subset of YFP-negative blood cells did react with the CD45 Ab (not shown). We have also previously shown that Tie1-YFP cells in circulation are negative for the phagocyte specific dye PKH-PCL26 [20]. We next examined whether adherent Tie1-positive cells were hematopoietic progenitor cells by labeling blood samples (HH12-20) with antibodies against chicken CD41, a marker of embryonic hematopoietic stem cells. Less than 1 percent of total blood-borne cells reacted with the CD41; while, the percentage of Tie1-YFP expressing cells, which reacted with CD41, is in the range of 0.5 to 1.3 percent.

To summarize we identified a subpopulation of hemoglobin negative, circulating Tie1-YFP-positive cells that can engage in substrate adhesion, and that were not leukocytes or macrophages. Further, such cells react strongly with QH1 antibodies and, therefore, were almost certainly vascular endothelial cells circulating in the bloodstream.

### Circulating Endothelial Cells Arise From Both Intra- and Extra-Embryonic Tissues

A current study suggests that all circulating endothelial cells (CECs) arise from extra-embryonic tissues and that intra-embryonic blood vessels cannot produce CECs [21]. However, we report here for the first time that Tie1-YFP endothelial cells can also enter circulation directly from primary intra-embryonic blood vessels (Movie S2, circle at 12.63 h; Movie S3, circles at 0–3.7 h, 5.5–6.6 h and 6.8–7.7 h). Recordings showed that Tie1 positive cells entered the bloodstream from both large and small vessels. Vessels in the head, the aortae and the sinus venosi as well as extra-embryonic vessels were all sources of Tie1-YFP circulating cells. For example, YFP cells were observed to shed from the dorsal aorta in Movie S2 (circles) and Movie S8. Other examples include cells that emerged from the forming sinus venosus in Movie S7 between approximately 9–12 h. Indeed, all wide-field recordings of Tie1-YFP embryos showed both individual cells and multi-cellular clumps entering the bloodstream from future great vessels (Movies S2, S3, S7, S8, S9).

Our ability to confirm that intra-embryonic mesoderm is also a source of circulating endothelial cells was complicated by two ambiguities. First, due to the recording frame rate we used, it was not possible to track individual cellular motion after the cell enters circulation. Second, it was possible that extra-embryonic mesodermal cells might have colonized the embryo proper, via cell immigration, prior to expression of Tie1-YFP and thus contributed to formation of intra-embryonic vessels. In order to mark intra-embryonic cells – but not extra-embryonic cells – we employed targeted fluorescent protein expression using a precision electroporation technique [22], [23]. The anterior-most primitive streak epiblastic epithelium (early Day 1) gives rise to intra-embryonic mesoderm that is highly vasculogenic [24], [25], [26]. Thus, by confining electroporation to a specific position in the HH4-epiblastic surface of the primitive streak it is possible to restrict, anatomically, which region of the mesoderm will eventually express an electroporated DNA plasmid. This approach is shown in Movie S4, with explanatory diagrams in Figure S1. In the present instance we used a DNA plasmid encoding fluorescent proteins to electroporate eleven specimens (Mito-YFP, n = 9; Mito-GFP; n = 1; and H2B-GFP, n = 1) [22], [23]. To confirm that the fluorescent protein plasmid transfected cells, which entered circulation, were endothelial cells and not hematopoietic cells, embryos were injected with Alexa 555-QH1 after electroporation. As mentioned, QH1 is a well-accepted quail endothelial cell marker.

**Figure 4:**
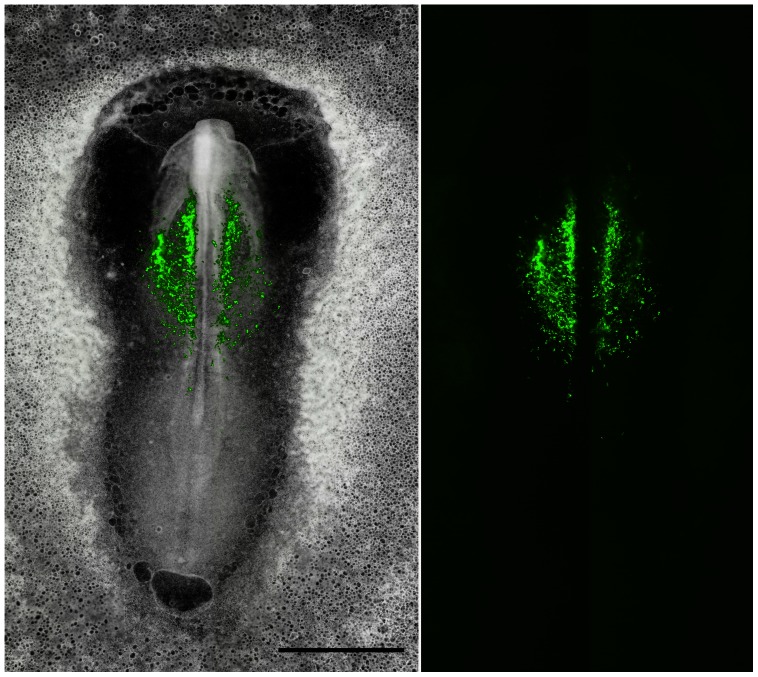
Precise electroporation, ex ovo, allows specific labeling of anterior intra-embryonic mesoderm. A montage of eight (8) XY microscope fields shows brightfield and epifluorescence images of a HH8 quail embryo that was electroporated approximately 10 hours earlier with Mito-YFP at stage HH4- (see Fig. S1 for details). The left panel shows the two optical modes superimposed. These data demonstrate that using our precision electroporation method it is possible to restrict all Mito-YFP labeling to intra-embryonic tissue exclusively. Specimens that displayed extra-embryonic fluorescence were discarded. Note that the bulk of the fluorescent cells are situated in a mesodermal compartment characterized by robust primary vasculogenesis. Using this targeted approach any Mito-YFP fluorescent cells observed in circulation after HH10 had to have originated from mesoderm of the embryo proper, i.e., did not arise from extra-embryonic mesoderm. Scale bar  = 1 mm or 1000 µm.

**Figure 5:**
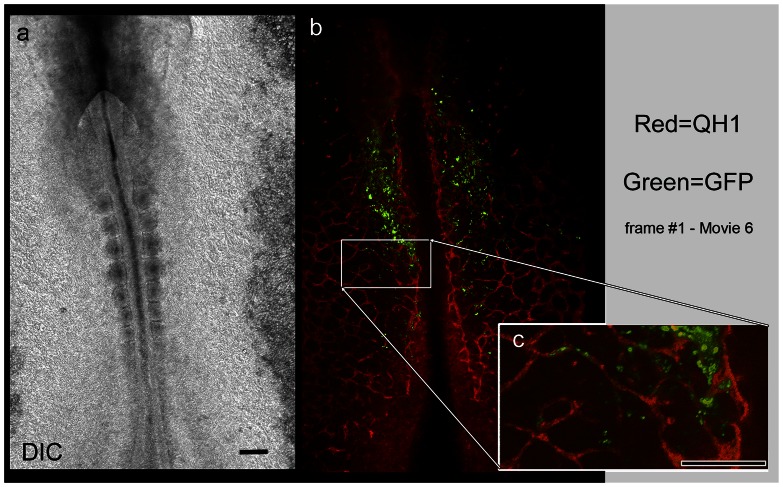
A sub-population of Mito-GFP labeled intra-embryonic mesoderm differentiates into endothelial cells and expresses the marker QH1. A DIC time-lapse image frame of a HH9 embryo (panel a), captured during a period of active vasculogenesis. The companion panel (b) shows the corresponding double fluorescence images indicating the presence of primary vascular networks. Red designates QH1 immunoreactivity and green designates Mito-GFP cells labeled by earlier (HH4-) electroporation. A region of interest (white box) is shown at higher magnification in Panel C. Note that at higher magnification there are cells within formed vascular elements that express both red and green fluorescence. Thus, some epiblastic cells electroporated hours earlier had differentiated into vascular endothelial cells at HH9 (QH1, red) when this time-lapse frame was captured. The time-lapse recording from which these images were extracted shows multiple Mito-GFP/QH1-positive cells entering circulation (see Movie S6 and Fig. 6). Note that, as expected, only a sub-population of the Mito-GFP mesoderm (green) differentiated into vascular endothelial cells and react with QH1 endothelial marker (red). Scale bars  = 100 µm.

**Figure 6:**
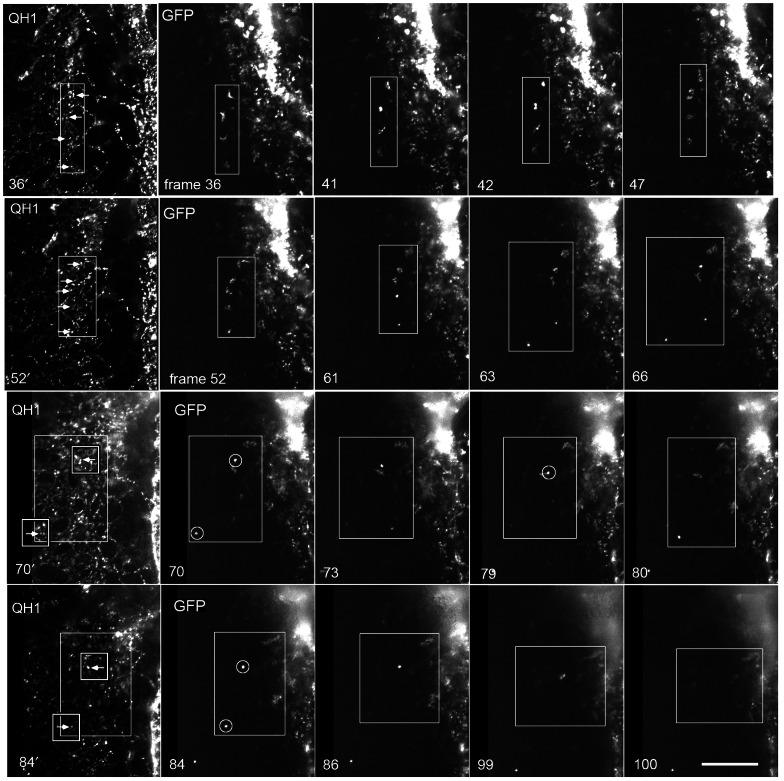
Double fluorescence time-lapse frames of QH1-positive cells and Mito-GFP labeled cells. All GFP-labeled cells were derived from electroporated intra-embryonic mesoderm shown in Figure 4. This figure is comprised of a series of image frames from a time-lapse recording of the embryo in Movie S6 (i.e., Fig. 5, Fig. 6 and Movie S6 are all the same specimen). Time points and regions of interest (ROI, white boxes) were selected to denote salient features. Each horizontal strip shows five images depicting four time points. The first and second frames in each strip depict the same time point acquired in both the red (QH1) and the green (GFP) optical channels, respectively (e.g., frames 36′ and frame 36). The succeeding three frames in the row show the Mito-GFP signal only. The white arrows denote QH1 positive cells in frame 36′ that correspond to the Mito-GFP positive cells in frame 36. During the time-lapse recording primordial endothelial cells shifted positions and engaged in cell division shown in frames 36-47. For example by frame 41, the second cell from the top (ROI) is condensed and rounded, suggesting possible cell replication, followed by cellular spreading (frames 42, 47). By the time frame 66 was captured all of the Mito-GFP cells in the ROI at frame 52 had moved and/or rounded up. Also, compared to earlier time points fluorescent cells in frame 66 are reduced in number indicating that one or more cells had departed the ROI. After frame 61 the cells of interest continued to move apart, compared to earlier time points; this is denoted by a larger ROI (white box). The GFP-labeled cells visible in frame 70 (circles) are QH1-positive (white boxes with arrows frame 70′). The encircled cell at the bottom of frame 70 is absent from the ROI approximately 13 minutes later (frame 73). In the interval between frames 79 and 80 a cell of interest (circle) was lost from its previous position. The same behavior is observed between frames 84 to 100. The QH1/GFP-positive cells are progressively lost from the ROI, such that by frame 100 all the original cells of interest (frame 36) are absent. Note the consistently bright cells at the periphery of the ROI; this shows that electroporated mesoderm maintains robust Mito-GFP expression throughout the recording. See Methods and Materials, and the Legend for Movie S6 regarding details on optics and image acquisition. Scale bar  = 100 µm.

Quail primitive streak cells were electroporated (HH4-). The resulting fluorescent cells, and their progeny, colonized the anterior-half of the intra-embryonic mesoderm at HH7 and 8 as shown in Movie S4, Figure 4, Figure 5, and Figure S1. Differential interference contrast (DIC) optics confirmed that the location of the fluorescent cells was confined to the embryo proper, i.e., area pellucida (Movie S4, Fig. 4, Fig. 5). Our ability to restrict expression of fluorescent proteins to intra-embryonic mesoderm confirmed the anatomical origin of any subsequent cells that expressed the fluorescent marker, or their progeny, during time-lapse recording (see Movies S4, S5, and S6). Wide-field time-lapse imaging of the electroporated embryos (n = 11) revealed significant numbers of fluorescent cells that engaged in rapid-displacement behavior and entered circulation. The recording in Movie S6 and the related images in Figure 6 show multiple examples of fluorescent cells that exited the ROI within a single frame (typically, 8-13 frames-per-hour, fph). The exposure time used for epi-fluorescence recordings was approximately 100 ms per frame. Recordings were made at multiple z-planes together with XY-stage control to capture several overlapping microscope fields of view (Methods). This allowed us to account for all cells in a ROI at all possible focal planes. On especially favorable occasions a circulating fluorescent cell was recorded during the brief interval the camera shutter was open (approximately 100 ms). In such cases the cell appeared as a ‘streak’, denoting a fluorescent object moving at high velocity in free circulation (circles, Movie S4 at 21.41 h–21.51 h; Movie S5 at 22.6–22.7 h).

The double fluorescence time-lapse image frames, in Figures 5 and 6, confirm the presence of numerous endothelial cells that express both Mito-GFP and Alexa 555-QH1. Movie S6 is the source of the panels displayed in Figure 5 and 6. The data also show that double-labeled cells were actively engaged in vasculogenesis at HH9 (Fig. 5b). Higher magnification (Fig. 5c) shows double-labeled cells that were manifestly residents of vascular endothelial cords/tubes. Indeed, all Mito-GFP cells present in vascular tubes/cords were also immunoreactive with QH1 antibody. Due to the non-lineage-specific nature of the Mito-GFP DNA plasmid, only a portion of electroporated mesodermal cells eventually differentiated into endothelial cells – thus Figure 5c shows numerous cells expressing Mito-GFP in their cytoplasm, which are not (and should not be) reactive with the QH1 cell surface antibody.

All electroporated embryos eventually showed fluorescent cells moving in the bloodstream of distant intra- and extra-embryonic vessels (Movies S4, S5). We concluded that endothelial cell rapid-displacement behavior occurred when doubly fluorescent cells (plasmid based fluorescence + QH1 immunoreactivity), which were constituents of endothelial tubes, entered circulation. Our anatomically-restricted electroporation experiments demonstrate unequivocally that avian intra-embryonic mesoderm is a source of circulating endothelial cells, albeit few in number as compared to extra-embryonic tissue.

### Behavior of Endothelial Cells Prior to Entering Circulation

These are the first empirical data ever produced showing primordial endothelial cells entering circulation in a warm-blooded animal (embryo), to the best of our knowledge. In fortuitous cases, playing the time-lapse recordings backward, frame-by-frame, provided insight into the location and behavior of individual endothelial cells minutes before such cells entered circulation (one frame interval). Mito-GFP/QH1-labeled cells displayed sharp, jerky, motion prior to leaving a ROI. One such ROI was delineated in four progressive filmstrips from a single recording (see Figure 6 which was extracted from Movie S6). Careful inspection of the ROI revealed that before entering circulation some cells ‘rounded-up’ and were abruptly transported a short distance to new positions. These behaviors occurred relatively quickly – often during a single time-lapse interval, in this case 9.2 minutes. In general, rapid displacement behavior was manifested as a cell that suddenly jumped or skipped to a nearby position, 20–50 micrometers distant, where it remained for several frames. A cell manifesting saltatory behavior sometimes remained within a local ROI; eventually, however, its abrupt motion behavior was followed by that cell's disappearance from the ROI, via circulation (see Introduction for operational definition of “saltatory behavior”).

In a few cases just before rapid displacement behavior occurred a Mito-GFP cell appeared to undergo cell division. An extended or protrusive cell of interest would round up and become brighter, followed by the appearance of a pair of cells. On rare occasions one of the two cells entered circulation while the other cell remained in the ROI; one such example is shown in Movie S6 at frames 7.7 hr–7.9 hr, which corresponds to panels 61 and 63 in Figure 6. These observations suggest that rounded-up post-mitotic endothelial cells may be more susceptible to the forces of fluid flow compared to more squamous adherent cells. Indeed, rapid displacement motion, after mitosis, was also observed when Tie1-YFP cells were transplanted to wild-type host embryos; for example see the circled ‘double nuclei’, in Frame 9.68 h of Movie S11, one of which is swept away by blood flow at Frame 10.03 h.

### Some Tie1-positive cells exhibit slow or ‘rolling’ displacement behaviors

In contrast to fluorescent cells moving swiftly in the bloodstream a different and equally striking activity was observed in transgenic embryos – namely, a ‘rolling’ behavior that was exhibited by intra-luminal Tie1-YFP endothelial cells. Close inspection to all Tie1-YFP wide-field recordings revealed the presence of endothelial cells juxtaposed to the vessel wall moving at speeds easily captured by slow frame rates of 6-12fph (Movie S9, arrowheads; Movie S3, circles at 0 h–3.7 h, 5.5 h–6.6 h, and 6.8 h–7.7 h). To confirm the rolling behavior at high spatial resolution, the phenomenon was recorded within one confocal plane in Movie S10. The confocal time-lapse data demonstrated a cell (red arrow) moving in the direction of flow, but at an exceedingly slow speed compared to cells in free circulation – which appear as fluorescent green streaks (yellow arrowheads). Similarly, the DIC recording in Movie S9 shows that ‘rolling’ fluorescent cells move far more slowly compared to RBCs streaming through the vasculature, which are readily visible in the DIC panel of Movie S9. Moreover, a rolling cell's proximity to the vessel wall suggested physical contact and interaction with the luminal face of the endothelium. Clusters, as well as individual cells, engaged in the slow ‘rolling’ behavior. For example, Movie S7 shows a high resolution ROI in which multiple clumps appear to roll along the lumen of the nascent sinus venous/vitelline vein. Movie S9 shows clusters and individual Tie1-YFP cells rolling and interacting with the luminal endothelium. Perhaps this behavior is most conspicuously exhibited by cells and cellular aggregates slowly ‘sloughing off’ the paired dorsal aortae in Movies S2 (circles) and during the first half of Movie S8.

### Endothelial Cell Transplantation Studies

**Figure 7:**
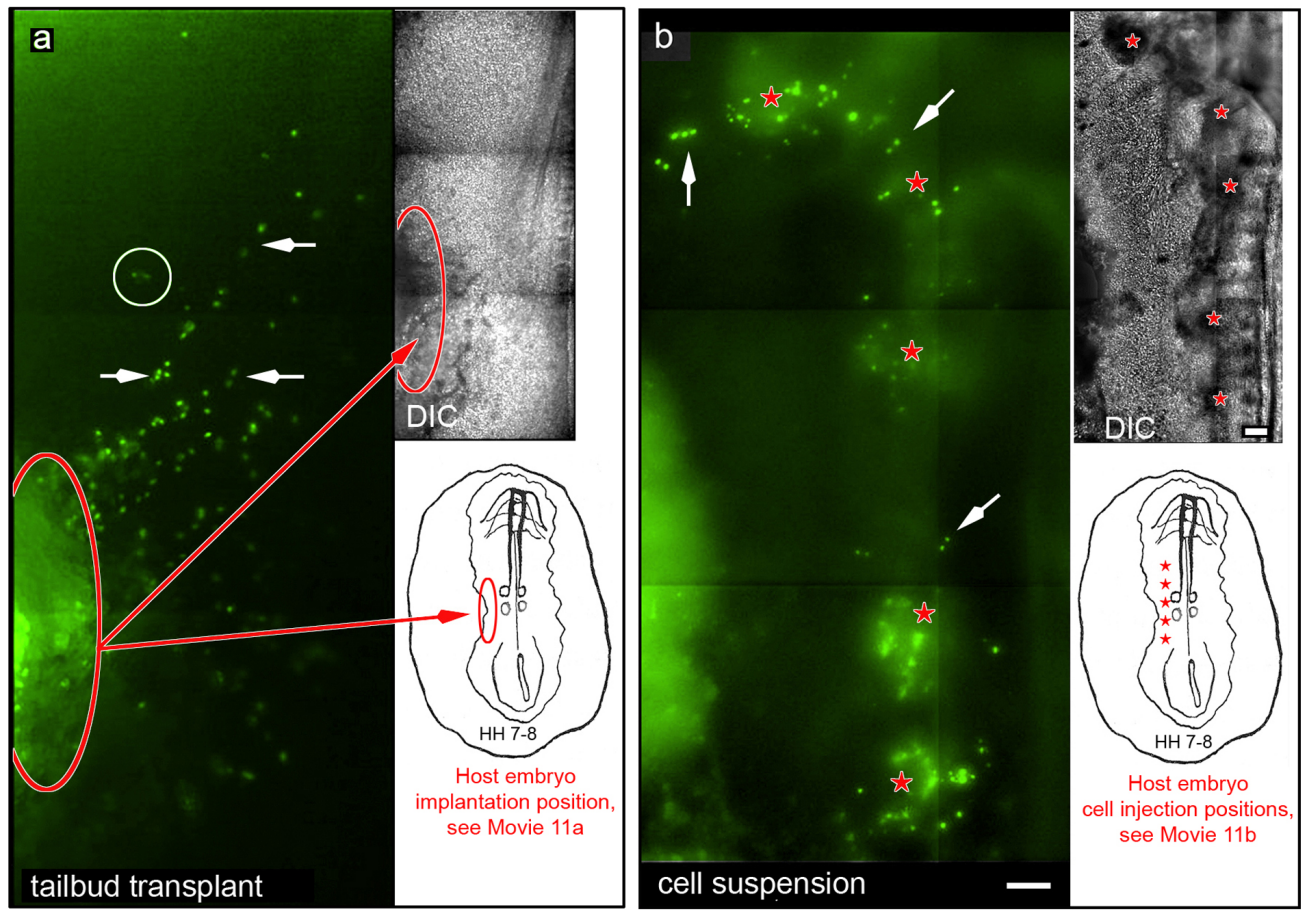
Transplantation of Tie1-YFP tissue or cells into wild type embryos show that donor cells are capable of engaging in vasculogenesis and entering circulation. The figure shows a single time point selected from two different time-lapse recordings. Both recordings are contained in Movie S11. **Panel (a)** shows Tie1-YFP tail bud tissue transplanted into a wild-type embryo (n = 6) in which fluorescent cells (green) have emigrated from the explant (red oval) and colonized host vascular elements (white arrows) in 4 out of 6 explants. The white circle indicates a pair of circulating cells that are lost from the ROI in the next frame (see Movie S11a at the 15.46 hr time point). Careful inspection of the Movie S11 reveals other Tie1-YFP endothelial cells that also entered circulation. The red oval in the diagram denotes the initial position of the transplanted tail bud tissue, before recording began. **Panel (b)** shows a wild-type embryo injected with a bolus of a disperse cell suspension isolated from HH10 Tie1-YFP embryos (n = 4). The image frame shown is extracted from Movie S11b at the 17.46 hr time point. Multiple injections were made parallel to the vertebral axis as denoted by the red stars in the DIC panel and the diagram. Most fluorescent endothelial cells (green) remained near the injection site (red stars). Many YFP cells, however, participated in vessel formation and joined the host vasculature (white arrows). Eventually, a few Tie1-YFP cells entered circulation (see the circled cells in Movie S11b). These data depicting transplanted tissue or cells demonstrate that donor Tie1-YFP endothelial cells are capable of participating in primary vasculogenesis and entering circulation when transferred to a host embryo. The line drawings in Panels 6a and 6b represent an idealized HH7-8 embryo. See the legend for Movie S11 regarding optics and image acquisition. Scale bars  = 100 µm.

To address the potential fate of circulating Tie1-positive cells using a different approach we conducted tissue, cell and blood transplantation experiments (n = 35). Tie1-YFP tail-bud tissues (e.g., similar to the area encircled in Fig. 3) were transplanted into wild type quail embryos (n = 6) lateral to the somites at HH6-8 (red ovals, Fig. 7a). Bright fluorescence continued to be expressed in donor tissue during the next 24 hours of time-lapse recording. In four out of six (4/6) implantation specimens Tie1-YFP cells initiated robust motility, emigrated from the host tissue and participated in local vasculogenesis, i.e., the cells formed vascular elements and integrated with host endothelial tubes/cords (arrows Fig. 7a; Movie S11a). Significantly, the vasculogenic behavior was followed by small numbers of fluorescent cells entering circulation (circle, Fig. 7a; circles, Movie S11a). Further, all donor Tie1-YFP cells (or their progeny) react with the QH1 antibody in chicken host embryos confirming an endothelial phenotype (n = 1, data not shown). The recordings demonstrated that Tie1-YFP/QH1 endothelial cells, which emigrated from transplanted tail-bud tissue, displayed vasculogenic, rapid displacement and saltatory behavior (arrows, Fig. 6.; circles, Movie S11a).

Two transplanted tail buds (2/7) did not shed Tie1-YFP cells into circulation. In these specimens the injected Tie1-YFP tissue remained fluorescent, formed a compact ‘ball’ or clump, but did not manifest detectable levels of donor cell locomotion or emigration into the host embryo. Thus, there appears to be a causal relationship between the initial degree of donor cell(s) motile activity and whether we subsequently observed locomotion, vasculogenic activity and shedding behavior.

Transplantation of mono-disperse ‘single’ cell suspensions (n = 8, 4 in quail and 4 in chicken hosts) was also conducted (Fig. 7b, Movie S11b). This approach tested whether Tie1-YFP cells, devoid of adhesions to the ECM and other cells, would enter circulation more efficiently compared to cells embedded in a coherent tissue. The donor preparation contained mono-disperse cell suspensions, 10-50% of which were Tie1-YFP positive. Multiple boluses of suspended cells, visualized by DIC optics, were injected into the mesodermal compartment of HH5-8 host embryos (red stars, Fig. 7b; Movie S11b). At each injection site a cohort of Tie1-YFP endothelial cells initiated motility and incorporated into host vascular tubes/DIC, (arrows, Fig. 7b); more importantly, the images in Movie S11b (circles) show fluorescent donor cells engaging in rapid displacement behavior and circulating through host vessels. Therefore, both tail bud transplants and cell suspensions yield a small number of fluorescent cells that entered circulation (as shown in Movie S11a at 14.87 h and 15.46 h (circles). All Tie1-YFP donor cells in chicken hosts were QH1 positive (not shown). We found no evidence that detaching resident Tie1-YFP-positive cells from their native adhesions increased the probability that such ‘single’ cells would eventually enter the bloodstream of a host embryo; if there was a trend, the data suggested that tissue explants integrated more vigorously with host vasculature compared to dispersed cells.

In a final series of transplantation experiments circulating Tie1-YFP cells were collected from the blood of transgenic quail (HH12-13) and transferred to the mesodermal compartment of either HH6-9 wild-type quail (n = 10) or chicken embryos (n = 10). Tie1-YFP fluorescent cells were evident over a 20–24 hr recording period. Nine out of ten transplants into chicken (9/10), and seven of ten specimens (7/10) in quail hosts, displayed Tie1-YFP endothelial cell motility. In rare instances we observed examples of rapid displacement behavior. Surprisingly, we were not able to detect circulating cells that expressed normal levels of Tie1-YFP fluorescence. Occasionally, we detected circulating cells with very weak YFP signal intensities, i.e., marginally above the level of thermal noise inherent in a cooled CCD camera (data not shown).

To summarize the transplantation experiments: 1) YFP tail-bud transplants either displayed no cellular outgrowth, or donor endothelial cells readily emigrated, engaged in robust vasculogenesis and produced circulating endothelial cells; 2) transplanted YFP endothelial cell suspensions uniformly produced motile vasculogenic cells which occasionally entered circulation; and 3) YFP donor cells isolated from blood displayed modest motility, rarely engaged in rapid displacement behavior, and did not produce detectable circulating cells in the host bloodstream.

## Discussion

### Positional fate versus lineage fate of circulating endothelial cells

We studied the origin of circulating endothelial cells using two complementary approaches. First, germline transformed tie1-H2B-YFP quail embryos were subjected to time-lapse imaging, with and without, in vivo QH1 fluorescence labeling. The presence of the Tie1 transgene insures that all members of the vascular endothelial lineage express this fluorescent differentiation marker [18], [27]. Time-lapse recordings of pre-circulation Tie1-YFP transgenic embryos showed hundreds of ‘stationary’ cells, present in endothelial tubes or cords, entering circulation. None of the circulating QH1-positive/Tie1-positive endothelial cells reacted with benzidine. However, we observed a significant sub-population of weakly fluorescent Tie1-YFP cells, which were QH1-negative. All members of the latter sub-population reacted with benzidine.

Our time-lapse images showed Tie1-YFP cells entering from both intra-embryonic and extra-embryonic vessels. The Tie1-YFP movies cannot prove, however, an‘intra-embryonic’ positional origin for any of the circulating endothelial cells. It is possible that all Tie1 positive cells entering circulation in the embryo proper become erythroblasts since the endothelium is known to have hemogenic potential [28], [29]. Furthermore, it is also possible that extra-embryonic endothelial progenitors were born, entered circulation, arrested in the intra-embryonic vasculature, and only then expressed the differentiation marker Tie1-YFP. In other words, the transgenic embryo data prove lineage fate (arising from vascular endothelium) but not positional fate.

The second approach entails spatially restricted electroporation to exclusively label intra-embryonic tissue – namely, the anterio-lateral intra-embryonic mesoderm. Simultaneous injection of fluorescent QH1 antibody meant that doubly fluorescent cells were endothelial cells. By such means, QH1-reactive intra-embryonic endothelial cells in tubes/cords could be traced backward in time to progenitor cells in the pre-gastrulation epiblastic epithelium. This observation shows unequivocally that intra-embryonic QH1-positive cells can also enter into blood circulation and that this behavior is not limited to extra-embryonic vessels.

Together, the two data sets prove that there is a small, but reproducibly observable, population of intra-embryonic primordial endothelial cells that enter circulation. This behavior is by no means restricted to any one anatomical region of the embryo, or any one portion of the vasculature. QH1-reactive, Tie1-YFP-positive, endothelial tubes from all segments of the vasculature give rise to circulating endothelial cells, including the lateral vascular network, aortae, endocardium, sinus venosi, extra-embryonic vessels and so forth. The origin of circulating endothelial cells from intra-embryonic mesoderm was an unexpected and novel result. The current literature indicates that only extra-embryonic vessels are capable of producing circulating endothelial cells [21]. Our studies were performed on embryos at a much earlier stage of development than previous work and this may account for the apparent discrepancy in results. The fact that we were the first to use dynamic imaging (on amniotes) at these stages uniquely allowed detection of rapid displacement behavior within the embryo proper. Furthermore, previous studies used chimeras of quail embryos (without a yolk sac) sutured to chicken embryos (with their yolk sac), a markedly different system compared to an intact embryo. Our results also strongly suggest that hemodynamics play a role in the entry of cells into circulation, it may be that the experimental chimeras do not achieve normal blood flow velocities characteristic of the stages studied here. Because we did observe that circulating Tie1-positive cells could differentiate into erythroblasts – we cannot rule out the possibility that cells entering from intra-embryonic vessels would eventually lose QH1/Tie expression and differentiate into erythroblasts.

### Circulating endothelial cell behavior

Virtually all DIC recordings show restricted fluid flow or physical bottlenecks in the primitive vascular network where Tie1-YFP cells entering circulation are temporarily trapped prior to flowing freely in the blood. Playing the movies backwards shows no apparent differences in the protrusive/motile activity of endothelial cells that eventually enter circulation, versus nearby endothelial cells, i.e., the cells of interest appear to be functionally differentiated endothelial cells. As mentioned above, when describing Figure 6, some presumptive circulating endothelial cells round up, and appear to engage in mitosis, before entering circulation (white box, Movie S6). The time-lapse data show scores, if not hundreds, of Tie1-YFP cells entering circulation. These two vastly different physical fates, i.e., remaining resident vessel cells or becoming blood-borne, blurs the definition of what constitutes a mature endothelial cell. We observed cells that can change shape, enter circulation and possibly modify their lineage fate. The epithelial-to-circulation behavior suggests that differentiated endothelial cells may be more phenotypically versatile than previously appreciated.

### Speculation on the lineage fate of circulating endothelial cells

It is tempting to speculate that upon entering circulation embryonic endothelial cells might trans-differentiate and thus gradually lose Tie1-YFP expression. The QH1-positive/Tie1-YFP cells, which are capable of adherence, may remain vascular endothelial cells and colonize distant vascular beds. Alternatively such cells may be capable of changing fate to other mesodermal lineages such as myeloid or smooth muscle cells [30], [31], [32]. The preponderance of circulating Tie1-YFP cells appeared to be slowing losing their fluorescence and acquiring an erythoblastic phenotype, based on reactivity with benzidine. Investigating the fate of circulating Tie1-YFP endothelial cells at stages beyond HH 14-16 is complicated by several issues: loss of the YFP signal, diminished optical clarity due to the markedly more massive (thick) embryos and higher intrinsic fluorescence noise levels. All these issues result in poor signal-to-noise ratios that preclude time-lapse recording and dynamic fate mapping of later stage entire embryos.

### Conclusions

Our most important findings, based on two distinct lines of fluorescence time-lapse experimental evidence are that: 1) the intra-embryonic mesoderm of a warm-blooded embryo gives rise to circulating endothelial cells; and 2) at early embryonic stages a remarkable proportion of total blood cells are Tie1-positive (up to 30%). We also report the first in vivo data showing the behavior of endothelial cells as they enter circulation in an amniote embryo. The data suggest the possibility that mitosis could make endothelial cells susceptible to entering circulation. Though our experiments were conducted using avian embryos, we strongly suspect that similar processes occur in all amniotes, including mammals.

## Materials and Methods

### Ethics Statement

According to NIH-USPHS and USDA guidelines, early avian embryos (fertile eggs), prior to mid-gestation, are not considered vertebrate animals for purposes of experimentation.

### Germ-line transformed Tie1-GFP reporter quail embryos

The generation and characterization of the *Tie1* transgenic quail lines Tg(*tie1*:H2B-eYFP) were described in detail [18].

### Culture of embryos on the microscope stage

The procedures for cultivation and electroporation of Japanese quail (*Coturnix coturnix japonica*, Ozark Egg Co., Stover, MO) have been described extensively [23], [33]. The embryos were maintained *ex ovo* by mounting the specimens on paper rings, making sure that the vitelline membrane remains intact. The embryos were washed free of yolk and placed in custom-made culture dishes [23], [33]. Embryos were placed ventral side up for injecting with antibodies, and for time-lapse recording after transfer to the environmentally controlled stage of a conventional upright microscope [33], [34], [35], [36].

### Electroporation of whole-mounted embryos

Japanese quail eggs were incubated until HH stage 3–4 and whole mounted embryos were prepared as described above. An approximately 0.2 µl bolus of DNA plasmid solution (1.5 µg/ul) was injected between the epiblast and vitelline membrane and electroporated using platinum wire electrodes at a precise anatomical position [23], [33], also see Figure S1. Electroporated specimens were screened for expression of GFP/YFP/RFP using a fluorescence stereomicroscope – specimens were inspected to ensure that no fluorescent signal was detected in *extra-embryonic* tissues. Upon reaching HH6-7 embryos were transferred to the microscope stage. In some cases, previously electroporated HH7-8 embryos were subsequently microinjected with Alexa™ 555-conjugated QH1 antibodies before time-lapse recording. The QH1 antibody is a standard marker of quail endothelial cells and their immediate progenitors [37].

### Wide-field time-lapse recording

Long-term, wide-field, time-lapse recordings of quail embryos (n = 105) were performed using an automated microscopy system (Movies, S1, S2, S3, S4, S5, S6, S7, S9, and S11); the instrumentation and methods were described in detail elsewhere [34], [35], [36]. Typically twelve-bit gray-scale images were recorded every 5–12 minutes in both DIC and epi-fluorescence modes (see individual Movie Legends). Images were acquired at 7-9 focal planes, separated by 10 µm, and in most cases at multiple XY positions arranged as overlapping image tiles. This procedure over-sampled the specimen in X-Y-Z space thus ensuring that any point of interest was in focus despite the normal movements of the embryo over an extended recording period (20–30 hours). The full thickness imaging approach also insures that no visible object (cell) can leave one focal plane, move out of focus, and be lost to observation.

The imaging system was based on a computer-controlled standard compound Leica DMR upright microscope equipped with a motorized stage and a QImaging Retiga-SRV™ camera, using a 5X or 10X Neofluar™ objective. The instrumentation for recording tissue-sized areas (1 mmx1 mm) at multiple focal planes and multiple illumination modes, at 1 µm resolution (10× objective), has been in use over ten years [35], [36]. Freely available, open source software code of our design (TiLa, KUMC) was used to process, align, smoothen and register the adjacent XY image tiles, from algorithm-selected Z planes, to produce a full-scale composite image for each time point (i.e., one movie frame). Depending on the size of the area to be imaged the number of XY tile images ranged from one (e.g., Movie S1) to eight (Movie S2). The resulting sequential image frames were concatenated and compiled as a movie file using ImageJ (NIH), then converted to a QuickTime™ file. Specimens are viewed from the ventral side using an upright microscope, thus the anatomical right of an embryo appears on the left in the recordings.

### Compression of wide-field time-lapse (KU/TiLa) image files

Full-resolution unprocessed time-lapse image sequences for a single experimental specimen can exceed 30 GB. An essentially ‘lossless’ compression algorithm is then used for archival purposes to produce a corresponding, high-resolution movie file that can exceed 5 GB. In order to reduce the archived file size to manageable level for publication, a second compression algorithm was used that results in a slight, but noticeable, loss of image detail. High-resolution (archived) versions of the Movie files included in this article are available upon email request to the corresponding author.

### Acquisition of confocal time-lapse images, Movies S8 and S10

In the case of confocal Movie S8 endothelial cell exfoliation from the aortae was imaged using embryos (n = 4) mounted in an environmental chamber on a Zeiss 510 META NLO™ confocal microscope equipped with a plan-Neofluar™ 20X/0.5 objective [18]. In the case of confocal Movie S10, TIE1-YFP reporter quail eggs were incubated at 38°C for 45–48 hours until the embryos reached HH12. Embryos were then cultured ex ovo as described [18], [38], [39], placed on a heated microscope stage and imaged using a Zeiss LSM Exciter™ confocal microscope, with a plan-Neofluar™ 20×/0.5 objective. Images were captured by ZEN™ software every 3 minutes.

### Preparation of cell suspensions from Tie1-YFP embryos

Ten HH10 Tie1-YFP embryos were collected [33] and trimmed of extra-embryonic tissue, then subjected to a series of trituration procedures, centrifugation and collagenase treatments. The resulting cellular pellet was drawn into a glass micropipette and introduced into the mesodermal space of HH5-8 wild type quail or chicken embryos (n = 20).

### Blood Draw/Counting Method

Blood samples from HH14 to HH18 embryos were obtained using a sharpened glass micropipette with a bore diameter of approximately 20 µm similar to the method describe earlier [23] except that samples were diluted immediately into cold heat-inactivated chicken serum and plated onto a glass hemocytometer. For the purposes of DIC and epifluorescence imaging we used a Leica DMRXA2™ microscope equipped with a Neofluar™ 20×/0.5NA objective lens. QCapture™ software was used to acquire images on a Retiga™ SRV camera at full resolution with 1×1 binning. Tie1-YFP fluorescence was captured using a 2000 msec exposure on all blood specimens to allow comparison between samples. ImageJ software was used to view images for counting YFP fluorescent nuclei. Total cell counts were obtained from DIC images. To count YFP nuclei in a consistent manner the epifluorescence image contrast setting was adjusted to the same values for all samples. To ensure a random sampling, each hemocytometer field was selected using DIC optics and then switched to epifluorescence to acquire the cell count data (fluorescent Tie1-YFP nuclei). Similar methods were used for harvesting HH12-13 embryo blood, however the sample volumes were far smaller and not sufficient for hemocytometer-based analysis.

### Benzidine Staining

Blood was drawn from embryos as described and resuspended in 100 μl D-MEM. Then 12 μl of suspended cells were pipetted onto a hemacytometer and immediately imaged in brightfield (BF) and Tie-1 (YFP) fluorescence using a 20× objective lens. The remaining 88 μl cell suspension was centrifuged for 5 minutes. The pellet was resuspended in 80 μl of fresh 0.05% benzidine/0.1 M sodium acetate solution. 0.4 μl of 15% hydrogen peroxide was added to the cell suspension. 12 μl was loaded on a hemacytometer and again imaged for both BF and YFP.

## Supporting Information

Figure S1 A scheme for restricting electroporation to anterior intra-embryonic mesoderm. The diagrams depict the electroporation protocol used for the time-lapse analysis as shown in Figures 4, 5 and 6 and Movies S4, S5 and S6. The drawing of an idealized HH4- embryo shows the position of the electroporation target in the primitive streak. The presumptive mesodermal cells (epiblastic) were electroporated with a DNA plasmid encoding either a nuclear (Movies S3 and S4) or a mitochondrial-directed fluorescent protein (Figures 4, 5, and 6; Movie S5). Electroporation of embryos at the position shown ensured that all cells, which later express fluorescence, are restricted to anterior intra-embryonic tissue. In other words the labeling strategy is designed to ensure that no *extra-embryonic* mesodermal progenitors are electroporated. The drawing of a one somite, HH7, embryo is based on time-lapse epifluorescence data (e.g., Movies S4 and S5) and depicts the expected fluorescent protein expression pattern in the anterior lateral plate mesoderm, approximately 10 hours after electroporation. Note that the expression pattern is well within the area pellucida, i.e., is restricted to the embryo proper (aip = anterior intestinal portal). A drawing of a HH8 embryo depicts the tissue field expected to contain H2B-GFP- or Mito-YFP-tagged mesoderm. The shaded ‘fluorescent’ region is restricted to a tissue domain within which intra-embryonic vasculogenesis takes place. This drawing corresponds closely to the fluorescently labeled specimen shown in Figure 4, and the embryo recorded in Movie S6. Scale bar  = 100 µm.
(TIF)

Movie S1 A time-lapse recording made at one frame per second (fps) in a local Region of Interest (ROI). The ROI is an area (825×675 µm) that was extracted from a wide-field recording, showing an extra-embryonic capillary bed in a HH 16 Tie1-YFP embryo. This movie demonstrates a number of endothelial cell behaviors. The streaks that appear to move at very high speed are YFP tagged endothelial cells moving at the rate of blood flow. Other fluorescent cells or small cellular clusters move at variable rates, many in a saltatory fashion characterized by rapid, sequential, starts-and-stops. The white circles at the 10.8-to-10.9 time-point highlight the journey of a cluster of Tie1-YFP endothelial cells percolating through the ROI. Note that the encircled cluster moves through the vascular bed slowly compared to freely flowing blood; further, the cluster appears to become trapped in bottlenecks as it courses through the small bore lumens. It is useful to stop the recording and manually advance frame-by-frame using the QuickTime™ software controls in order to observe events of interest. Playing the recording backwards also facilitates comprehension of the various behaviors. The recording rate is 59 frames per minute or about 1.0 second between frames. The compression algorithms used in creating this movie result in a loss of resolution compared to the native image files (see Methods). Mag bar  = 100 µm.
(MOV)

Movie S2 A wide-field recording of a Tie1-H2B-YFP embryo from HH7 to HH14. The fluorescence signal shows numerous examples of endothelial cells moving in an abrupt saltatory fashion compared to the majority of ‘non-motile’ cells situated in vascular tubes. An observer perceives the motion as a Tie1-YFP cell (nucleus) ‘jumping’ to a new location within one time-lapse frame. Such motion requires a displacement speed that is far greater than cell autonomous (‘self-propelled’) locomotion. The saltatory behavior is best visualized by advancing the recording frame-by-frame using the QuickTime™ software controls. Perhaps more importantly, there are numerous examples where a Tie1-YFP cell is present in one frame and is lost from view during the next recording cycle, this event is operationally defined here as “rapid displacement behavior”. Numerous examples are visible in the ROI denoted by the white box. We interpret these empirical data as evidence that a given cell-of-interest entered circulation and was swept away by fluid flow. Tie1-YFP cellular aggregates also display rapid displacement behavior (white box). In a variation of rapid displacement behavior cell clusters are observed shedding from the luminal face of large vessels such as aortic vessels (circles 12.37 h–24.21 h). Furthermore, throughout the later time points there are scores of Tie1-YFP clusters circulating freely through the great vessels (22–29 h recording interval). The DIC and fluorescence Movie S2 frames are montages of eight XY image fields. The recording rate is approximately 7.5 frames per hour (fph) or 8 min between frames. The compression algorithms used in creating this movie result in a loss of resolution compared to the native image files (see Methods). Mag bar = 100 µm.
(MOV)

Movie S3 A wide-field recording, and two ROI panels at higher resolution, depicting events in a HH10 to HH18 Tie1-H2B-YFP embryo (see Fig. 3). The optical data represent three orders of spatial magnitude (µm-mm). The white boxes correspond to a ROI depicted in the panel to the right at higher magnification. The boxed area shows morphogenesis of the right (anatomical) vitelline artery. Single cells (nuclei) and clusters of Tie1-YFP cells can be seen moving with a wide variety of speeds compared to ‘stationary’ endothelial tubes (encircled nuclei, 0.0–3.7 h, 5.5–6.6 h, 6.8–7.7 h). Note the large aggregates of fluorescent cells in the tail bud region, which dissipate as the recording progresses (10.0 h large circle, left panel). As 17.0 h approaches hundreds of Tie1-YFP cells and cellular aggregates are observed flowing from caudal positions toward the nascent inflow tract of the heart as indicated by the white chevron. These empirical data show multiple examples of rapid displacement behavior. The recordings strongly suggest that large numbers of Tie1-YFP cellular aggregates shear off from the tail bud and enter systemic circulation. The recording rate is 9.1fph or 6.6 min between frames. The compression algorithms used in creating this movie result in a loss of resolution compared to the native image files (see Methods). Mag bars  = 100 µm.
(MOV)

Movie S4 A recording of an embryo electroporated with a plasmid encoding a H2B-GFP construct and the corresponding DIC data. The H2B-GFP plasmid was electroporated such that only intra-embryonic mesoderm expresses the GFP. The wide-field optics confirm that plasmid-derived nuclear fluorescence is restricted to intra-embryonic, and only intra-embryonic mesoderm; i.e., compare the fluorescently labelled areas with the corresponding areas in the brightfield (BF) image. Nuclear-directed green fluorescence is detectable from HH Stage 5 until the end of the recording at 22.6 h. Cells derived from the primitive epiblast (HH4-) are observed to gastrulate and form the splanchnic mesoderm of the head and trunk (also see Fig. 4 and Fig. S1 for details). Some of the H2B-GFP labelled mesodermal cells are specified to the endothelial lineage and engage in vasculogenesis. A few labelled endothelial cells eventually enter circulation (circle at 21.41-21.51 h). These data confirm that cells derived from electroporated *intra*-embryonic mesoderm give rise to circulating cells – thus confirming the cell's positional fate. The data do not, however, establish an endothelial lineage fate for the circulating cell (i.e., the fluorescent marker is not endothelial lineage specific). The compression algorithms used in creating this movie result in a loss of resolution compared to the native image files (see Methods). Mag bar  = 200 µm.
(MOV)

Movie S5 A recording of an epiblastic stage embryo (HH4-) electroporated with a DNA plasmid encoding H2B-GFP. As with the specimen above (Movie S4) the H2B-GFP plasmid was electroporated such that only intra-embryonic mesoderm expresses the GFP, as confirmed by comparing the fluorescence recording with the corresponding DIC recording. The middle panel shows a projection of five (5) Z-axis focal planes at the 22.6 hour time point, while the right panel shows the individual image captured at each focal plane. Notably this recording shows fluorescent cells flowing in the blood designated by arrowheads. The time interval for acquiring each focal plane (Z-step) is approximately 100 msec, which fortuitously corresponded to the transit of a blood-borne cell-of-interest (arrowhead) as it flowed from a head vessel into the sinus venosus. The recording rate is 4.6 fph or 13 min between frames. The compression algorithms used in creating this movie result in a loss of resolution compared to the native image files. Mag bars  = 200 µm.
(MOV)

Movie S6 A recording of an epiblastic stage embryo (HH4-) electroporated with a DNA plasmid encoding a mitochrondrial-directed yellow fluorescent protein (Mito-YFP). The Mito-YFP plasmid was electroporated such that only intra-embryonic mesoderm of the embryo proper was fluorescently tagged (see Movie S4, Fig. 4 and Fig. S1). The ROI delineated by the white box shows a number of Mito-YFP cells engaged in vasculogenesis (Fig. 6). The live specimen was also microinjected with QH1 antibody to confirm the endothelial identity of selected cells shown in the ROI (see the arrows in Fig. 6). Note that most of the cells in the ROI (white box) are abruptly lost from view as time progresses, suggesting the cells engaged in rapid displacement behavior and entered circulation. Figure 6 shows 16 key individual frames extracted from this Movie. Thus, this, and similar recordings include simultaneous positional fate (YFP) and lineage fate (QH1) data. This embryo, the specimens shown in Movies S3, S4, and other time-lapse recordings not shown demonstrate that plasmid tagged intra-embryonic tissue gives rise to circulating cells (n = 11). Further, in all cases examined such electroporated cells are reactive with the endothelial specific QH1 antibody. The recording rate is 7.5fph or 8 min between frames. The compression algorithms used in creating this movie result in a loss of resolution compared to the native image files (see Methods). Mag bar  = 100 µm.
(MOV)

Movie S7 A wide-field and high magnification time-lapse recording of a Tie1-YFP quail embryo (DIC) from HH9 to HH13. The ROI (white boxes) delineates the presumptive sinus venosus and inflow tract of the heart at progressively higher resolution. The recordings show that clusters of Tie1-YFP endothelial cells are swept into this region and slowly ‘tumble’ along the nascent endothelial tubes leading toward the heart. A given cluster of circulating endothelial cells will move at variable speeds and will intermittently become ‘trapped’ depending on the size of an endothelial cluster and the bore-size (caliber) of a given vessel. Individual resident endothelial cells engage in rapid displacement behavior before being swept away by blood flow, showing that circulating endothelial cells emerge from venous vessels. The recording rate is 4.8fph or 12.3 min between frames. The compression algorithms used in creating this movie result in a loss of resolution compared to the native image files (see Methods). DIC  =  differential interference contrast. Mag bars  = 100 µm.
(MOV)

Movie S8 A recording made on a laser scanning confocal microscope depicting one image plane. The paired dorsal aortae and the associated medial-most elements of the lateral vascular network are shown beginning at approximately HH13. The pseudo-colored green fluorescence denotes Tie1-YFP endothelial cells. Note that the endothelial cells that comprise the aortic tubes ‘slough-off’ and enter circulation. The newly dislodged cells tend to remain in close association with the luminal face of the aortic endothelium, reminiscent of leukocyte rolling behavior, before being swept away by blood flow. Other circulating Tie1-YFP endothelial cells appear to originate from lateral arteries feeding the aortae. These data demonstrate that circulating endothelial cells emerge from both aortic and arterial branch vessels. A confocal image frame was captured approximately every 11 mins or 5.4fph for 13 hrs. Mag bar  = 100 µm.
(MOV)

Movie S9 A recording demonstrating ‘rolling’ behavior in the lumens of vitelline artery tributaries A number of endothelial cell nuclei are denoted with arrowheads. Early in the movie a few marked cells display an exceedingly slow rolling behavior compared to the speed of RBC as judged by DIC optics. The Tie1-YFP cells appear to be in contact with the luminal face of the arterial endothelium – reminiscent of leukocyte rolling behavior in mature vascular beds. Eventually the presumptive rolling cells are swept away by blood flow. Other Tie1-YFP cells marked by arrowheads appear to vibrate within a local cell-sized domain before abruptly departing the ROI via circulation. Some marked cells display a combination of the two behaviors. At the end of the recording all cells designated by arrowheads have entered circulation. The compression algorithms used in creating this movie result in a loss of resolution compared to the native image resolution (see Methods). This 2 hr Movie was made using a recording rate of 2.1 frames per minute or 28.6 seconds between frames. Mag bar  = 100 µm.
(MOV)

Movie S10 A confocal time-lapse recording of a Tie1-YFP vascular bed at high magnification with one frame taken every three minutes. The thin optical section shows the outlines of a capillary sized network. The red arrow denotes a single Tie1-YFP endothelial cell moving at approximately 0.04 µm per second whereas blood flows at about 1 mm/sec [40]. The motion of the marked cell is characteristic of rolling behavior similar to that observed during the homing of leukocytes and ‘mature’ circulating endothelial cells to sites of vascularization. Comet-like streaks (yellow arrowheads) denote the path of Tie1-YFP cells moving at the nominal speed of a RBC. Mag bar  = 50 µm.
(MOV)

Movie S11 Two separate donor/host transplantation experimental recordings combined into a single movie. Explanatory diagrams are found in Figure 7. **Panel a.** This recording (panel a) shows tail bud tissue that was removed from a Tie1-YFP transgenic embryo and then implanted into the mesodermal space of a wild-type quail embryo. The data show that fluorescent endothelial cells emigrated from the Tie1-YFP donor tissue and participated in host vasculogenesis (also see Fig. 7a). The movie shows numerous Tie1-YFP cells that were engaged in rapid displacement behavior and/or moving freely in the host bloodstream (circles, 13.69–16.13 h). One cell engaged in “rapid displacement” behavior, designated by a circle at 16.13 h, may have integrated into a host vessel after first exiting the tail bud transplant at 13.69 h. This tissue transplantation experiment was recorded using 5.0 min between frames or 11.9fph. The movie frame at interval 15.46 hours is the image in Figure 7a. **Panel b.** In this experiment several aliquots of a dispersed cell suspension, prepared from a HH10 Tie1-YFP embryo, were transplanted, via injection, into a wild-type embryo. Fluorescent donor cells emigrated from the local injection site (DIC) and appeared to participate in host vasculogenesis (also see Fig. 7b). A few Tie1-YFP cells were visibly moving via blood circulation, while other fluorescent cells engage in rapid displacement behavior (circles). All Tie1-YFP explanted cells tested were reactive with the QH1 antibody (not shown). The cell transplant recording was made using 6.8 min between frames or 8.8fph. The movie frame at interval 17.56 hours is the image in Figure 7b. The compression algorithms used in creating this movie result in a loss of resolution compared to the native image resolution (see Methods). Mag bars  = 100 µm.
(MOV)
