# Supplementary figures and images for: Embryogenesis of the First Circulating Endothelial Cells
Source: PLoS One. 2013 May 30;8(5):e60841. doi: 10.1371/journal.pone.0060841 (PMC3667859; doi:10.1371/journal.pone.0060841)

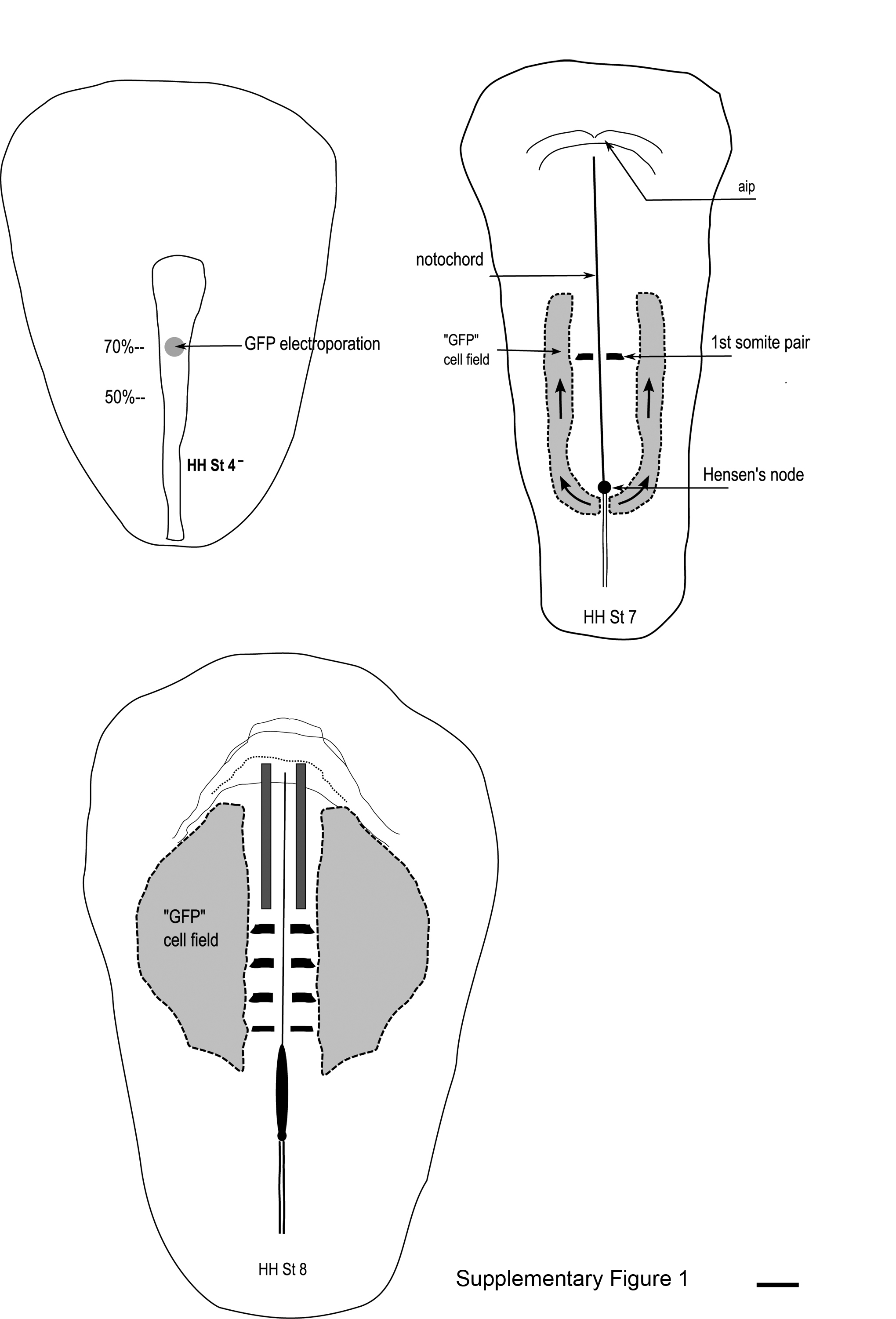

Supplement: Figure S1 — A scheme for restricting electroporation to anterior intra-embryonic mesoderm. The diagrams depict the electroporation protocol used for the time-lapse analysis as shown in Figures 4, 5 and 6 and Movies S4, S5 and S6. The drawing of an idealized HH4- embryo shows the position of the electroporation target in the primitive streak. The presumptive mesodermal cells (epiblastic) were electroporated with a DNA plasmid encoding either a nuclear (Movies S3 and S4) or a mitochondrial-directed fluorescent protein (Figures 4, 5, and 6; Movie S5). Electroporation of embryos at the position shown ensured that all cells, which later express fluorescence, are restricted to anterior intra-embryonic tissue. In other words the labeling strategy is designed to ensure that no extra-embryonic mesodermal progenitors are electroporated. The drawing of a one somite, HH7, embryo is based on time-lapse epifluorescence data (e.g., Movies S4 and S5) and depicts the expected fluorescent protein expression pattern in the anterior lateral plate mesoderm, approximately 10 hours after electroporation. Note that the expression pattern is well within the area pellucida, i.e., is restricted to the embryo proper (aip = anterior intestinal portal). A drawing of a HH8 embryo depicts the tissue field expected to contain H2B-GFP- or Mito-YFP-tagged mesoderm. The shaded ‘fluorescent’ region is restricted to a tissue domain within which intra-embryonic vasculogenesis takes place. This drawing corresponds closely to the fluorescently labeled specimen shown in Figure 4, and the embryo recorded in Movie S6. Scale bar = 100 µm. (TIF) [file pone.0060841.s001.tif]
